# A Rare Ultra-Long-Term Complication of Occluder Recanalization Due to Spontaneous Perforation of Polyvinyl Alcohol Membrane of Atrial Septal Defect Occluder: A Case Report and Review of the Literature

**DOI:** 10.3389/fcvm.2022.926527

**Published:** 2022-07-22

**Authors:** Kun Xiang, Huanwei Zhuang, Qin Wu, Mi Tang, Jinfu Yang, Chengming Fan

**Affiliations:** Department of Cardiovascular Surgery, The Second Xiangya Hospital, Central South University, Changsha, China

**Keywords:** atrial septal defect, closure, occluder recanalization, ultra-long-term complication, surgery

## Abstract

Percutaneous closure of atrial septal defect (ASD) has emerged as a feasible alternative strategy to surgical repair in many cardiac centers worldwide. Occluder recanalization due to device failure is a rare and severe complication that often occurs within weeks to years after ASD closure. We reported a rare ultra-long-term complication of occluder recanalization due to delayed spontaneous perforation of polyvinyl alcohol (PVA) membrane of ASD occluder after 18 years of ASD closure. Surgical removal of the faulty device and reconstruction of the atrial septum with a bovine pericardial patch was performed. The patient was discharged and recovered uneventfully without syncope or residual shunt. The cause of this rare complication of spontaneous PVA membrane perforation of the occluder has not been fully detected. To our knowledge, this is the first report about PVA membrane perforation of an occluder that occurred soon after ASD closure.

## Background

Atrial septal defect (ASD) is one of the most common congenital heart diseases in children, accounting for 7–10% of all congenital heart diseases (1). Percutaneous closure of ASD has emerged as a viable alternative to surgery when feasible in the case of favorable anatomy (2–4). The technique of catheter intervention for the treatment of ASD and patent foramen ovale was pioneered by King and Mills in 1975, which led to the invention of various devices from the late 1980s to the mid-1990s (5). The mushroom umbrella occluder (Huayi Shengjie Co., Ltd., Beijing, China) is made of two disc frames woven by a nitinol wire mesh and has a mushroom-shaped hole structure with self-expanding properties. The two discs and the waist are covered with medical polymer PVA membrane film to enhance the occlusion effect. PVA is a water-soluble polymer, which becomes insoluble for medical applications with formaldehyde or glutaraldehyde cross-links (6). The PVA membranes have a long history of permanent implantation in the medical field (2–4, 7). However, the complications of spontaneous perforation of the PVA membrane have been observed with ASD occluders made by both domestic and foreign manufacturers. Neither the previous surgeons nor the manufacturers could explain the mechanism of the PVA membrane disappearance. To date, the reports of long-term complications of occluder recanalization due to spontaneous PVA membrane perforation are extremely rare (8). Herein, we reported a 40-year-old woman with an ultra-long-term complication of massive left-to-right shunt due to spontaneous PVA membrane perforation of the occluder after 18 years of ASD closure.

## Case Report

**Figure 1:**
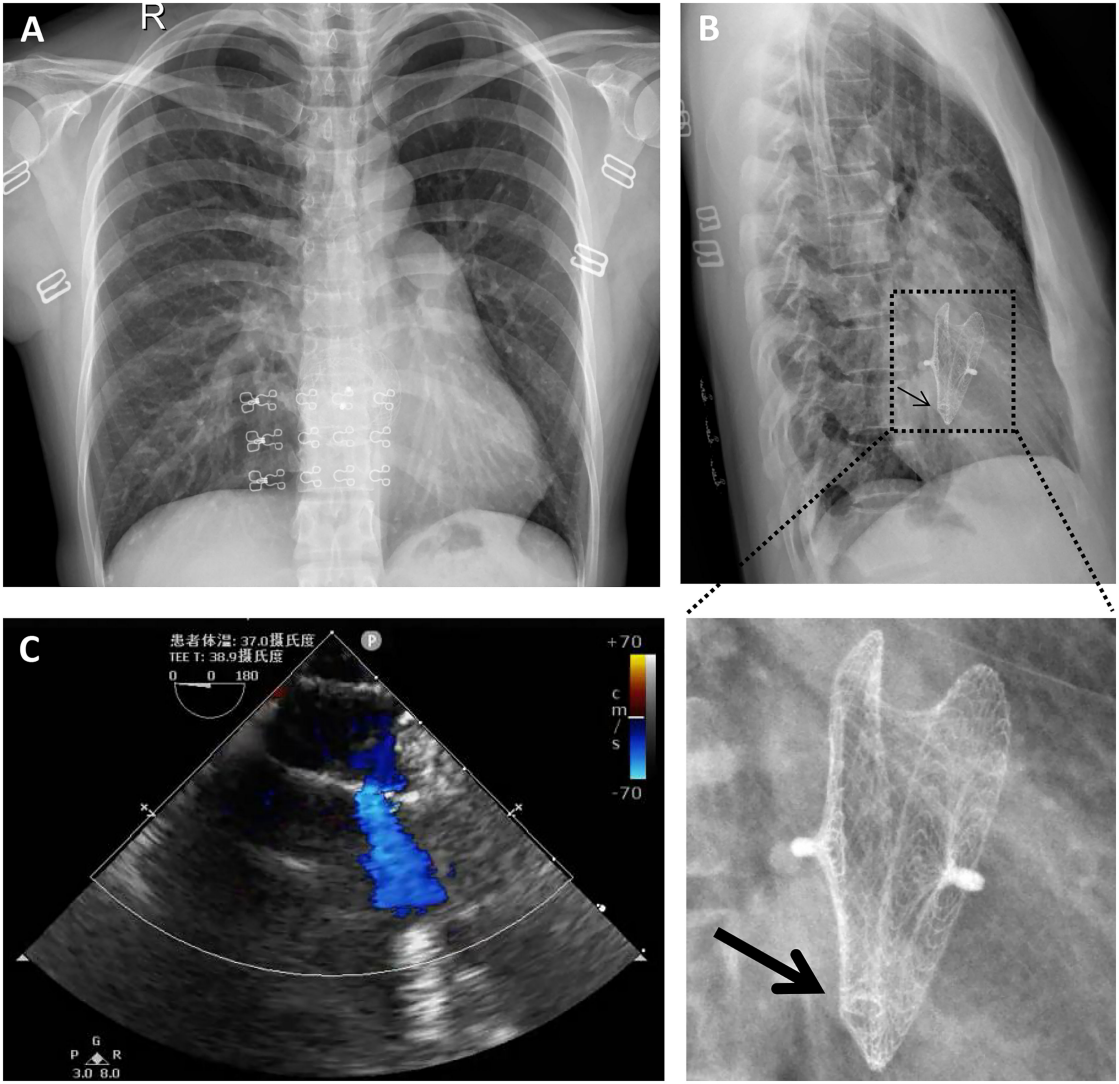
Chest radiography **(A,B)** and transthoracic echocardiography **(C)** preoperatively indicated that most parts of the closure were deformed, especially the left atrial side [**(B)**, arrow]; a significant multi-bundle left-to-right shunt through the device and a small adhesion on the occluder was detected **(C)**.

A 40-year-old female with sudden syncope was referred to our department. She has an 18-year history of percutaneous ASD (30 mm in diameter) closure with a 40-mm mushroom umbrella occluder (Huayi Shengjie Co., Ltd., Beijing, China). The surgical result was satisfactory, and no residual shunt was seen. Regular re-examination 3 years after the operation showed no abnormality. Two cesarean sections after 4 and 7 years of percutaneous ASD closure and a hysteromyoma surgery after 13 years of percutaneous ASD closure had been done. Transthoracic echocardiography and electrocardiogram during these three hospitalizations showed no abnormality. The patient in our case has not been followed up and had not undergone transthoracic echocardiography for the last 5 years. In the past 3 months, the patient had two episodes of sudden syncope, one was induced by exercise, and another had no obvious incentive, accompanied by sweating and pale complexion, which was relieved spontaneously in about 5 min. She denied chest pain, chest tightness, shortness of breathing, and palpitations. On physical examination, a soft blowing murmur was heard in the second and third costal margin of the left margin of the sternum. There were no other notable clinical findings during a physical examination and no medical, family, or psychosocial history including genetic information about cardiovascular disease. X-rays (Figures 1A,B) showed that most parts of the closure were deformed, especially the left atrial side (Figure 1B, arrow) and that the nitinol frame of the occluder was not displaced and there was no evidence of a frame fracture. The electrocardiogram demonstrated sinus rhythm and an incomplete right bundle branch block. Transthoracic echocardiography revealed a recurrent significant multi-bundle left-to-right shunt through the device and small adhesions were visible on the occluder (Figure 1C). Moreover, the enlarged right heart chambers and mild mitral valve regurgitation were detected. The patient was eventually diagnosed with a late-onset residual shunt of transcatheter device occlusion of ASD (occluder recanalization).

**Figure 2:**
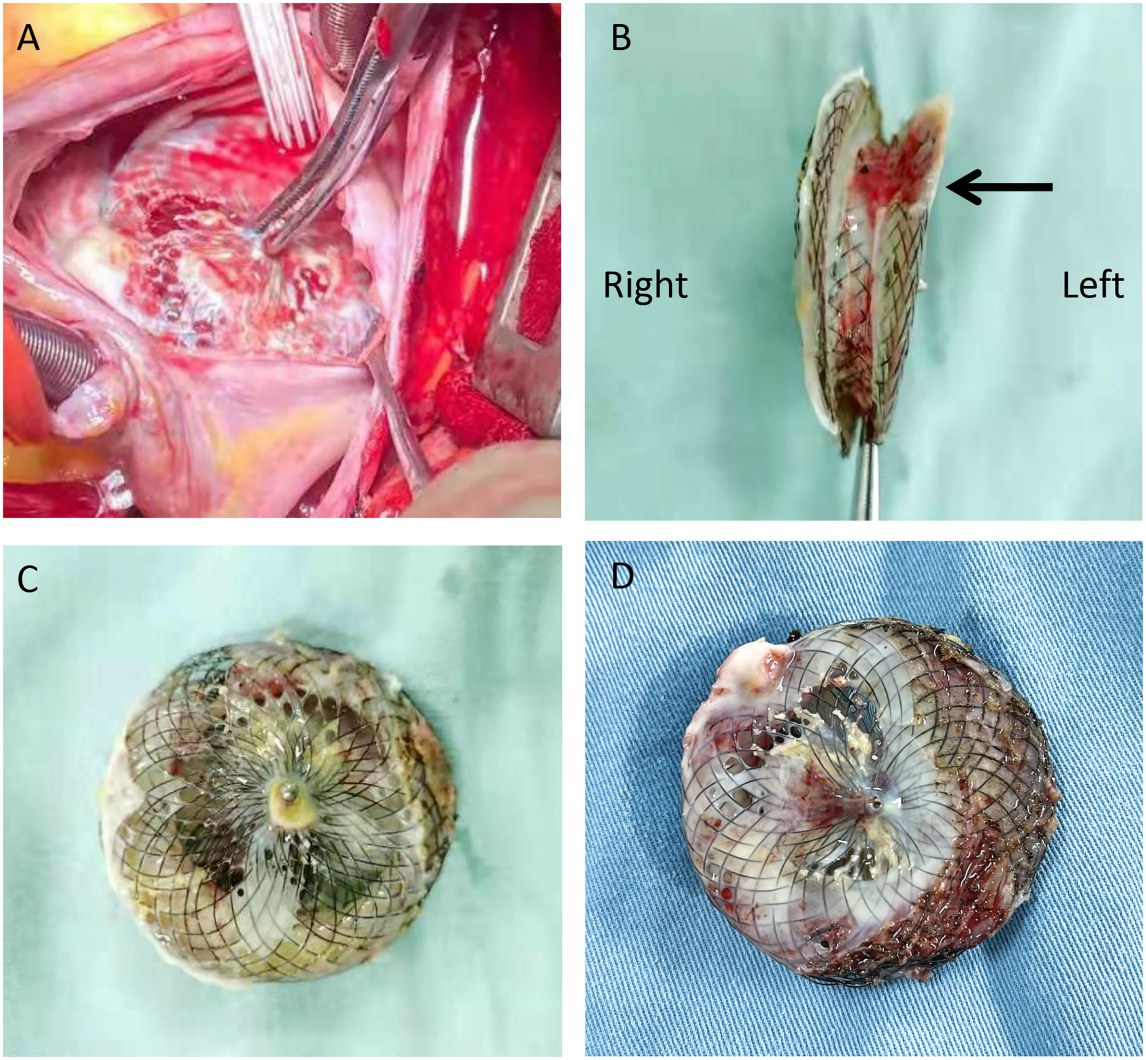
Intraoperative view of the procedure: After opening the right atrium, a deformed occluder was found in the atrial septum **(A)**; the left side of the occluder was damaged more obvious than the right [**(B)**, arrow]; The PVA membranes showed multiple perforations and were partly dissolved, and the diameter of the largest void was around 10 mm **(C,D)**.

**Figure 3:**
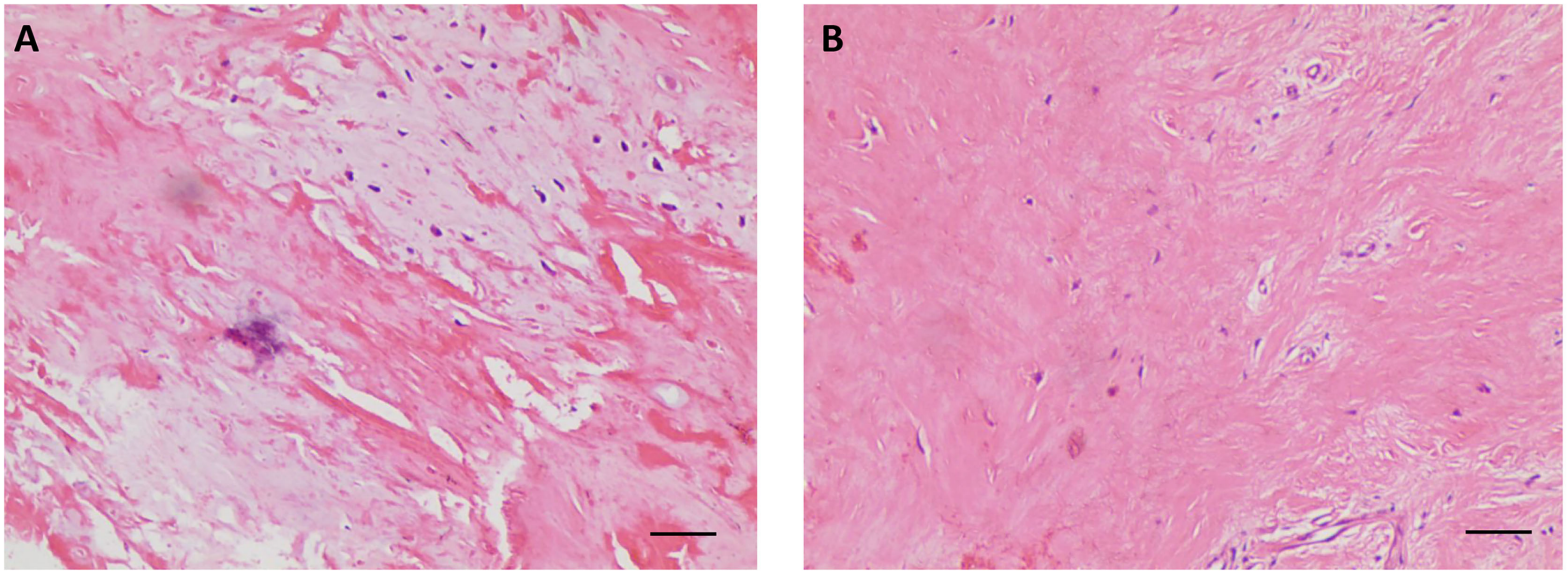
Postoperative histological examination with hematoxylin-eosin staining showing the proliferation of fibrous collagenous tissue **(A,B)**. (100 ×, scale bar = 50 μm).

**Figure 4:**
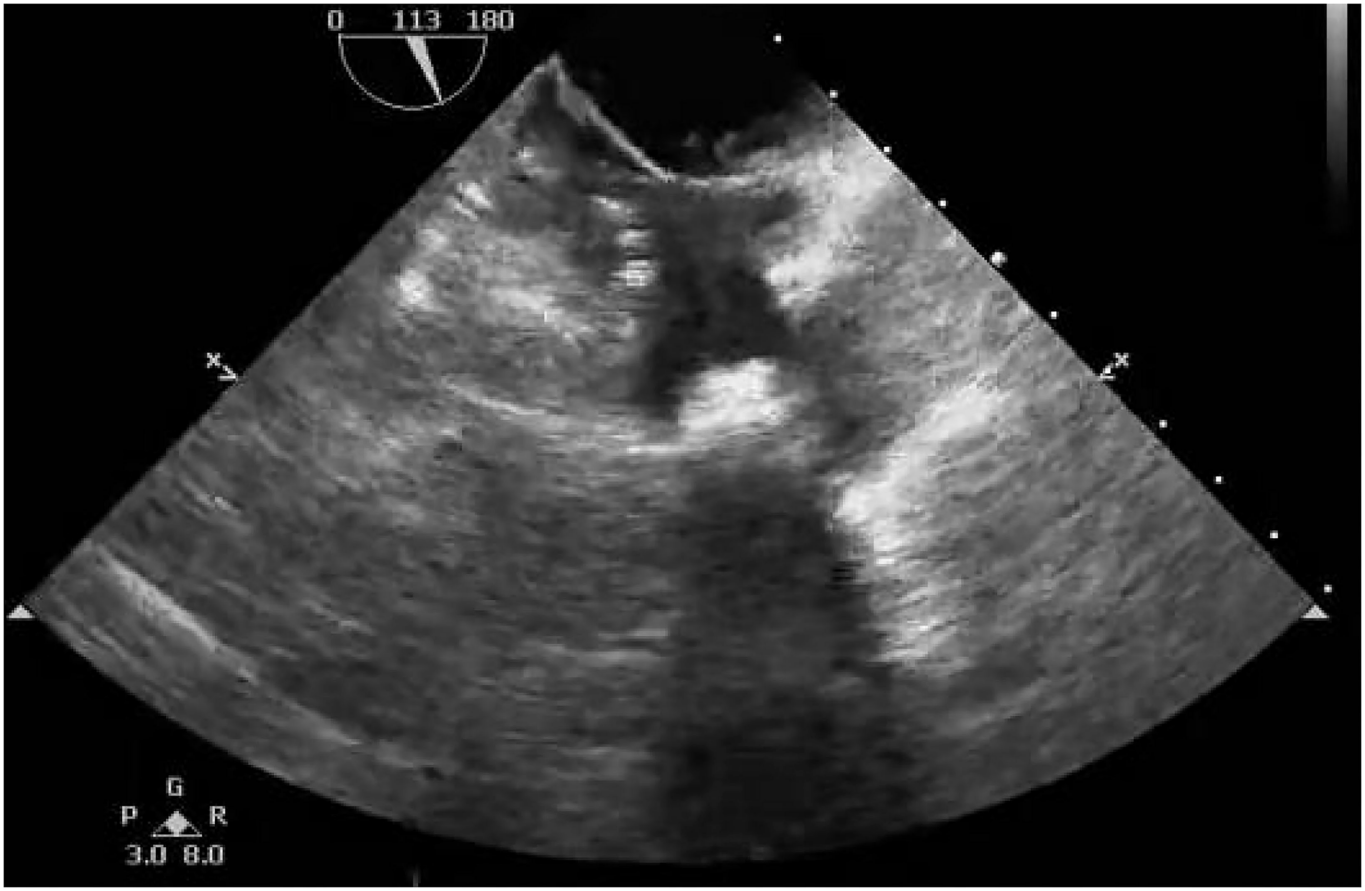
Postoperative transthoracic echocardiography showing that no residual shunt through the atrial septum was detected.

Considering the multiple perforations of the occluder with an unusual irregular surface of the discs (Figure 1B), and after a careful discussion with the patient and her family, an open heart surgery was scheduled and performed. A standard median sternotomy incision was performed. The aorta was then cannulated, followed by separate cannulas placed in the superior vena cava and inferior vena cava. After full heparinization, cardiopulmonary bypass (CPB) was routinely applied. The aorta was cross-clamped, and a cold cardioplegic solution (Del Nido) was instilled *via* the aortic root to arrest the heart. After the right atrium was opened, the initialized tissue of the failed occluder was sufficiently freed, the occluder was then released and completely removed, and a bovine pericardial patch was used to reconstruct the atrial septum. During the operation, most parts of the closure were deformed (Figure 2), especially the left atrial side closed to the edge of the pulmonary vein, which resulted in the possible cause of late-onset residual shunt. The PVA membranes showed multiple perforations and were partly dissolved, the diameter of the largest void was around 10 mm, and the left atrial side of the occluder was damaged more obvious than the right (Figure 2B, arrow). No thrombus and other vegetations were seen. Furthermore, according to the pathology report (Figure 3), the surface of the occluder has not been fully epithelialized, instead, there was an extensive proliferation of fibrous collagenous tissue. This may have been due to the constant flow of blood. The implanted device did not show evidence of infection, and the bacterial cultures were negative. Finally, the patient recovered uneventfully without recurrent syncope and residual shunt (Figure 4).

## Discussion

Percutaneous closure of ASD has emerged as a feasible alternative to surgical repair due to its shorter recovery and less invasiveness in the late 1990s and has even become the treatment of choice in many heart centers worldwide (9, 10). It is well-known that percutaneous ASD closure also has potential short-term problems, such as occluder displacement and dislodgement (11, 12), residual shunt, arrhythmia (13), occluder device failure (14), occluder abrasion (15), bleeding or thromboembolism (16), air embolism (17), hemolysis (18), pericardial effusion or cardiac tamponade (15), mitral regurgitation (19), headache or migraine, etc. There are also some interventional complications, such as anesthesia accident, wound infection, arteriovenous fistula, and so on. With the development of medical equipment and technology, percutaneous ASD closure is known to have satisfactory short-term outcomes and complication rates. However, an important consideration for percutaneous ASD closure is the long-term and ultra-long-term complications that have emerged after its widespread adoption. Some of these, including cardiac erosion (15, 20–23), infective endocarditis (24, 25), and thromboembolism (26, 27), carry unignored morbidity and mortality (4, 28). We report a case of spontaneous perforation of the PVA membrane observed after 18 years in a patient treated with a 40-mm mushroom umbrella occlude (Huayi Shengjie Co., Ltd., Beijing, China). Similar cases have been previously reported in many versions of the PVA membrane-covered ASD occluder (29–31) (Table 1). However, to our knowledge, this is the first report about the PVA membrane perforation of occluder occurring at the latest time after ASD closure.

**Table 1:** Summary of reported cases of polyvinyl alcohol membrane perforation of atrial septal occluders.

**First author (year)**	**Patient age/sex**	**Device type**	**Device size**	**Occluder recanalization after procedure**	**Symptom**	**Management**	**References**
Bozyel (2)	54/F	Cardia Ultrasept septal occluder	30 mm	2 years	No	Surgical device removal and Gore-Tex patch repair	(2)
Labombarda (3)	20/F	Ultrasept II ASD occluder device	20 mm	1 months	Recurrent dyspnea	Surgical device removal and Gore-Tex patch repair	(3)
Ramoglu (4)	4/M	Cardia Ultrasept II ASD occluder	20 mm	1 week	NA	Surgical device removal and Gore-Tex patch repair	(4)
Aubry et al. (7)	77/M	ASD Ultrasept II closure device	24 mm	4 months	No	Surgical device removal and Gore-Tex patch repair	(7)
Aubry et al. (7)	41/F	ASD Ultrasept II closure device	32 mm	3 months	No	Surgical device removal and Gore-Tex patch repair	(7)
Ten Freyhaus (8)	72/M	Atrial septal defect occluder system	NA	8 years	Worsening dyspnea on exertion	Surgical device removal and bovine pericardial patch repair	(8)
Chamie et al. (9)	28/F	Ultrasept^TM^ II CARDIA ASD occluder	20 mm	3 months	Easy tiredness and fatigue on exertion	Device-in-device technique	(9)
Chamie et al. (9)	33/F	Ultrasept^TM^ II CARDIA ASD occluder	16 mm	4 months	NA	Device-in-device technique	(9)
Chamie et al. (9)	49/F	Ultrasept^TM^ II CARDIA ASD occluder	16 mm	6 months	NA	Device-in-device technique	(9)
Chamie et al. (9)	17/F	Ultrasept^TM^ II CARDIA ASD occluder	14 mm	3 months	NA	Device-in-device technique	(9)
Bartel (29)	62/F	ATRIASEPT II device	24 mm	6 weeks	No	Surgical device removal and patch repair	(29)
Bartel (29)	42/F	ATRIASEPT II device	20 mm	5 weeks	No	Surgical device removal and patch repair	(29)
Kitamura (30)	20/M	Amplatzer septal occluder	NA	3 years	NA (infective endocarditis)	Surgical device removal and bovine pericardial patch repair	(30)
Weryński (31)	9/M	Cardia Ultrasept occluder	20 mm	4 years	NA	Surgical device removal and bovine pericardial patch repair	(31)
Weryński (31)	6/F	Cardia Ultrasept occluder	20 mm	3 years	NA	Surgical device removal and bovine pericardial patch repair	(31)
Aguiar Rosa (32)	39/F	Ultrasept ASD Occluder	22 mm	2 years	NA	Device-in-device technique	(32)
Bhattacharyya et al. (38)	69/M	Cardia Ultrasept septal occluder	28 mm	10 months	Several transient neurological events	Covering the damaged membrane with another occlusion device	(38)

*NA, Note available; M, Male; F, Female*.

The PVA is a synthetic polymer widely used in medical devices due to its good biocompatibility, chemical resistance, low adsorption to proteins, non-toxicity, and adhesion (6, 9, 32). Although the application of this material in ASD occluders has been generally successful, some cases of spontaneous disintegration and perforation of the PVA membrane have been described (Table 1). Neither the previous surgeons nor the manufacturers could explain the mechanism of the PVA membrane disappearance. It may be due to the incomplete endothelialization of the occluder, poor occluder placement, long-term medications, underlying immunocompromise, syndromic comorbidities, or metabolic disorders (2, 28, 32). Undoubtedly, more detailed and comprehensive follow-up data were needed to answer that question. In our patient, there was no evidence of an early and mid-term residual shunt due to device failure, but we cannot speculate on the specific cause of long-term device perforation. Similar complications of occluder recanalization due to dissolution of the PVA membrane has previously been reported as early as 1 week and as late as 8 years after implantation (4, 8). We report a case of spontaneous perforation of the PVA membrane of the occluder 18 years after surgery, so there should be no direct relationship between this complication and the time of device implantation. In our case, we did not find any evidence of infection, long-term medications, or systemic disease, so it is speculated that this may be the cause of PVA membrane degradation of the occluder, external force damage, or long-term endothelialization insufficiency. Another possible reason may be that the size of the occluder was too large, which make the endothelial tissue unsuitable for migration, and the endothelialization coverage was poor. Although complete endothelialization of ASD devices was thought to occur 3–6 months after device implantation, late incomplete endothelialization has been described based on animal and human studies (33–36). It can be seen from the disassembled occluder that the left atrial side near the edge of the pulmonary veins was more seriously damaged than the right. In addition, mitral valve regurgitation and long-term pulmonary venous blood flow scouring lead to damage of the PVA membrane of the occluder, followed by chronic dissolution. After some adverse events, the manufacturer announced the development of a new device in hopes of ameliorating this complication by adding a Gore-Tex patch between two metal disc pieces (3). The new device was proved to be safe and feasible. This conclusion was drawn from a study of 30 Mexican patients after ASD closure without occluder recanalization due to the PVA membrane perforation at follow-up for 6 (range: 1–15) months (37). However, the conclusion was only based on early follow-up data.

**Figure 5:**
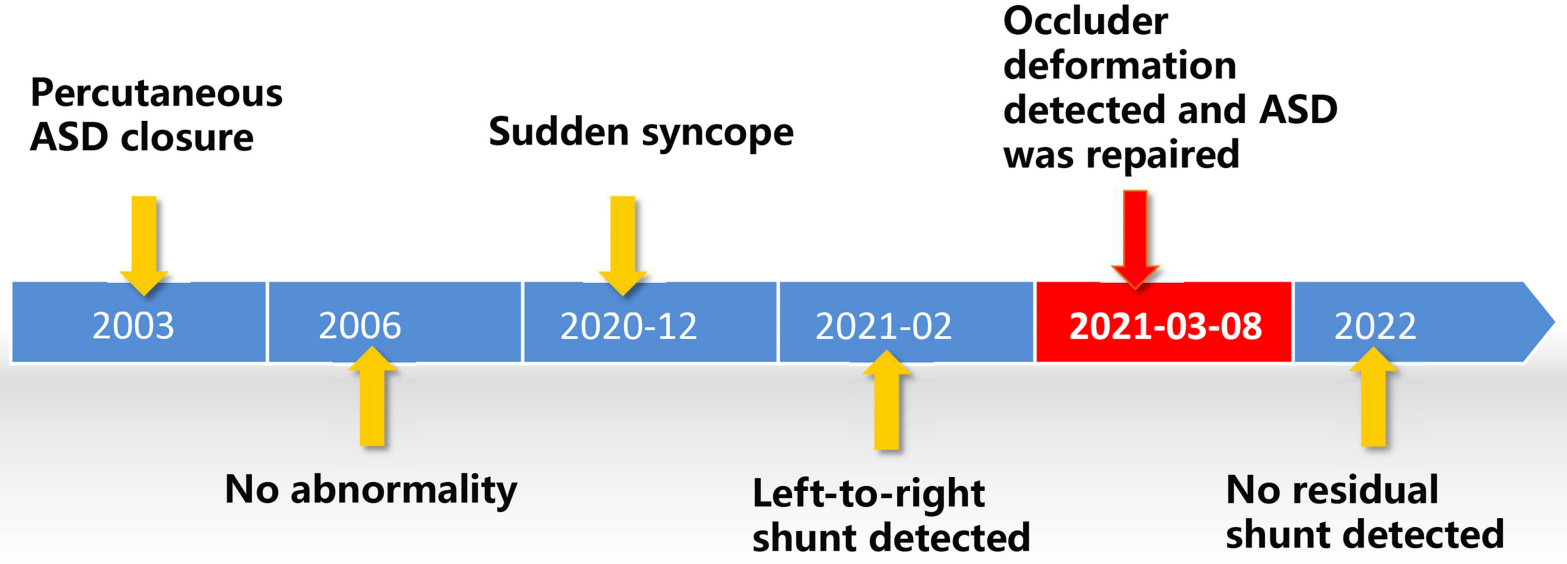
A figure showcasing a timeline with relevant data from the presented case.

The patient in the presentation first experienced syncope 18 years after ASD closure, but device perforation may have occurred months or years earlier. A timeline with relevant data from the presented case was shown in Figure 5. Patients with device failure were mostly asymptomatic but may also present with progressive dyspnea, fatigue, or stroke (3, 9, 38). It has been reported that occluder failure often induces some long-term complications after ASD closure, such as thrombosis on the surface of the occluder, thromboembolism, infective endocarditis, cardiac erosion, nickel hypersensitivity, and valve damage (8, 24, 25, 28). In most reported cases of occluder recanalization, surgical removal of the faulty device and repair with a patch were preferred (2). With the development of medical equipment and technology, covering the damaged membrane with a second device with intervention is considered a viable alternative to surgery when feasible in case of favorable anatomy (9, 32, 38). This should be done at a heart center with comprehensive imaging interventional equipment and experienced interventional cardiac surgeons.

## Conclusion

In conclusion, for the first time, we report a rare case of severe left-to-right shunt due to spontaneous perforation of the PVA membrane of ASD occluder 18 years after ASD closure. The cause of the rare serious complication of occluder recanalization due to the PVA membrane perforation has not been clearly explained. Surgeons and manufacturers should be aware of this potential ultra-long-term complication that cannot be ignored, and they should conduct long-term systematic follow-up examinations for all patients implanted with occluder devices to reduce the potential losses of the patient while obtaining comprehensive clinical data to guide the optimization of devices and technologies.

## Data Availability Statement

The raw data supporting the conclusions of this article will be made available by the authors, without undue reservation.

## Ethics Statement

The studies involving human participants were reviewed and approved by the Ethics Committee of the Second Xiangya Hospital of Central South University. The patients/participants provided their written informed consent to participate in this study.

## Author Contributions

KX drafted the manuscript. CF designed the study. HZ, QW, MT, CF, and JY revised the manuscript. KX, MT, and QW were responsible for the collection of data or analysis. All authors read and approved the final manuscript.

## Funding

This work was supported by the Key Project of Science and Technology of Hunan Province (No. 2020SK53420 to JY).

## Conflict of Interest

The authors declare that the research was conducted in the absence of any commercial or financial relationships that could be construed as a potential conflict of interest.

## Publisher's Note

All claims expressed in this article are solely those of the authors and do not necessarily represent those of their affiliated organizations, or those of the publisher, the editors and the reviewers. Any product that may be evaluated in this article, or claim that may be made by its manufacturer, is not guaranteed or endorsed by the publisher.
